# Efficacy and Safety of Rifaximin in the Prevention of Recurrent Episodes of Hepatic Encephalopathy: A Systematic Review and Meta-analysis

**DOI:** 10.5152/tjg.2023.22575

**Published:** 2023-06-01

**Authors:** Ahmed Elmoursi, Ahmed Taha Abdelsattar, Farag Khalil, Hosam Shabana, Hendawy Abdel-Moety Zedan, Ismail Mohamed El Mancy, Ibrahim Ghounim Ramadan, Sadek Mostafa

**Affiliations:** 1 University of Kentucky Faculty of Medicine, Lexington, Kentucky, USA; 2 Fayoum University Hospital, Fayoum, Egypt; 3 Department of Internal Medicine, Al Azhar University Faculty of Medicine, Cairo, Egypt

**Keywords:** Rifaximin, cirrhosis, encephalopathy, liver

## Abstract

**Background:**

Rifaximin is an oral antimicrobial drug with a broad-spectrum effect. It locally regulates the function and structure of intestinal bacteria and decreases intestinal endotoxemia. We aimed to investigate the preventive role of rifaximin in recurrent episodes of hepatic encephalopathy in cases with a history of hepatic diseases.

**Methods:**

We searched PubMed, Scopus, and Web of Science for the relevant studies using the following search strategy: “(Rifaximin) OR (Xifaxan) AND (cirrhosis) OR (encephalopathy).” We assessed the risk of bias using Cochrane’s risk of bias tool. We included the following outcomes: recurrence of hepatic encephalopathy, adverse events, mortality rate, and time to the first episode of hepatic encephalopathy from the time of randomization (days). We performed the analysis of homogeneous data under the fixed-effects model, while analysis of heterogeneous data was performed under the random-effects model.

**Results:**

We analyzed data obtained from 999 patients from 7 included trials. The overall risk ratio proved that the rifaximin group was associated with a lower recurrence rate than the control group (risk ratio [RR] = 0.61[0.50, 0.73], *P* = .001). We found no significant variation in both groups regarding adverse events (RR = 1.08 [0.89, 1.32], *P* = .41), and mortality rates (RR = 0.98 [0.61, 1.57], *P* = .93). The overall risk of bias results was low.

**Conclusion:**

The meta-analysis showed that in patients allocated to the rifaximin group, the incidence rate of hepatic encephalopathy was significantly lower when compared with those in the control group with no difference in both groups regarding adverse events and mortality rates.

**Main Points**

- Rifaximin locally acts on the gastrointestinal tract, regulating the function and the structure of intestinal bacteria and decreasing intestinal endotoxemia.
- Rifaximin significantly lowers the recurrence rates of hepatic encephalopathy compared with control.
- The adverse events were similar in both the rifaximin and control groups.
- Rifaximin is a well-tolerated drug that has a favorable profile.

## INTRODUCTION

Hepatic encephalopathy (HE) is considered one of the neurological disorders that result from the inability of the liver to prevent the accumulation of toxic substances in the blood.^1^ Chronic liver diseases may lead to liver cirrhosis, which is a pathological change in the liver structure (fibrogenesis and hepatocyte necrosis). Disturbed liver function, decompensated liver cirrhosis, and portal hypertension can cause many complications such as spontaneous bacterial peritonitis (SBP), ascites, hepatorenal syndrome (HRS), esophageal and gastric variceal bleeding, and HE.^2^ Hepatic encephalopathy negatively affects the quality of life of the patients as it causes deterioration of cognitive function and increases the frequency of falls.^3,4^

The accumulation of ammonium is the main cause of HE, which is why the treatments of HE are designed to accelerate the metabolism of ammonium or prevent the production and absorption of ammonium.^5,6^ The treatment of refractory ascites and recurrent variceal bleeding is transjugular-intrahepatic-portosystemic shunt (TIPS).^7,8^ The development of HE is one of the major complications after TIPS, especially during the first months.^9–11^ Not all cases of HE require hospitalization as the episodes of HE are usually mild.^12^

Many studies proved that some treatments are effective in HE such as nonabsorbable disaccharides, antibiotics, and l-ornithine l-aspartate. Nonabsorbable disaccharides such as lactulose cause decreasing intestinal absorption and production of ammonia.^6,13,14^

Rifaximin is an oral antimicrobial drug derived from rifamycin and has a broad-spectrum effect against gram-negative, anaerobic, and gram-positive enteric bacteria.^14^ Rifaximin is locally acting in the gastrointestinal tract.^15^ It regulates the function and the structure of intestinal bacteria and decreases intestinal endotoxemia.^16,17^ That is why rifaximin plays an important role in the protection of cirrhotic patients from SBP and recurrent episodes of HE.^14,18–20^

In this study, we aim to identify the role of rifaximin in the prevention of recurrent episodes of HE in patients with a history of liver diseases.

## Materials and Methods

We adhered to the Preferred Reporting Items for Systematic Reviews and Meta-analysis (PRISMA) guidelines and the Cochrane handbook of systematic review and meta-analysis of interventions^21,22^ while conducting this study.

### Electronic Search

We looked through 5 databases, Scopus, PubMed, and Web of Science for the relevant studies using the following search strategy: “(Rifaximin) OR (Xifaxan) AND (cirrhosis) OR (encephalopathy)”.

### Inclusion Criteria

All studies applied to these criteria were involved in our study:

1. *Participants*: Patients with a previous history of HE;
2. *Intervention*: rifaximin;
3. *Comparator*: any control;
4. *Outcomes*: recurrence of HE, adverse events, mortality rate, and time to the first episode of HE from the time of randomization (days);
5. *Study design*: randomized clinical trial.

### Exclusion Criteria

1. Observational studies and non-randomized trials;
2. Studies with no available full text;
3. Animal studies.

## Screening of Results

We imported the relevant studies from a systematic search of the databases to an Excel workbook^23^ using the EndNote X8.0.1 version. We conducted a 2-phase screening process according to the eligibility criteria. The title and abstract screening were the first step. Full-text screening was incorporated in the second one.

### Data Extraction

Following the screening process, we extracted data from 3 main categories: (1) general characters of the included studies and included patients such as age, gender, model for end-stage liver disease (MELD) score, and the number of HE episodes. (2) Data of the outcomes eligible for analysis including recurrence of HE, adverse events, mortality rate, and time to the first episode of HE from the time of randomization (days). (3) Data for the main domains of quality assessment according to Cochrane’s risk of bias tool.^24^

### Statistical Analysis

We performed our analysis using Review Manager Software (RevMan 5.4.1). We had dichotomous outcomes, so we performed our analysis using event and total. Also, we had continuous outcomes, so we performed our analysis using mean and SD. For heterogeneous outcomes, a random-effects model was used, while homogeneous data were analyzed using a fixed-effects model; using the Chi-square tests and *I* ^2^ index to assess the heterogeneity.^25^ Any values of *I* ^2^ > 50% or *P* <.1 were considered heterogeneous. We tried Cochrane’s leave-one-out method to resolve the heterogeneous outcomes.^25^

### Quality Assessment

Quality assessment of this meta-analysis was performed using the guidelines of the Grading of Recommendations, Assessment, Development, and Evaluations (GRADE). All included studies were clinical trials. We performed the quality assessment using Cochrane’s risk of bias tool.^24^ This tool comprises the following domains: blinding allocation of the included patients into each group, proper randomization, blinding of both personnel and participants (double-blinding), blinding of patients only (single-blinding), or not blinding at all, attrition bias, selection bias (outcomes reported matches with that of the protocol or not), awareness of the outcome assessor (whether blinded or not), and other bias. The total risk of bias for the studies has been assessed as well.

## RESULTS

### Summary of Included Studies

**Figure 1.**
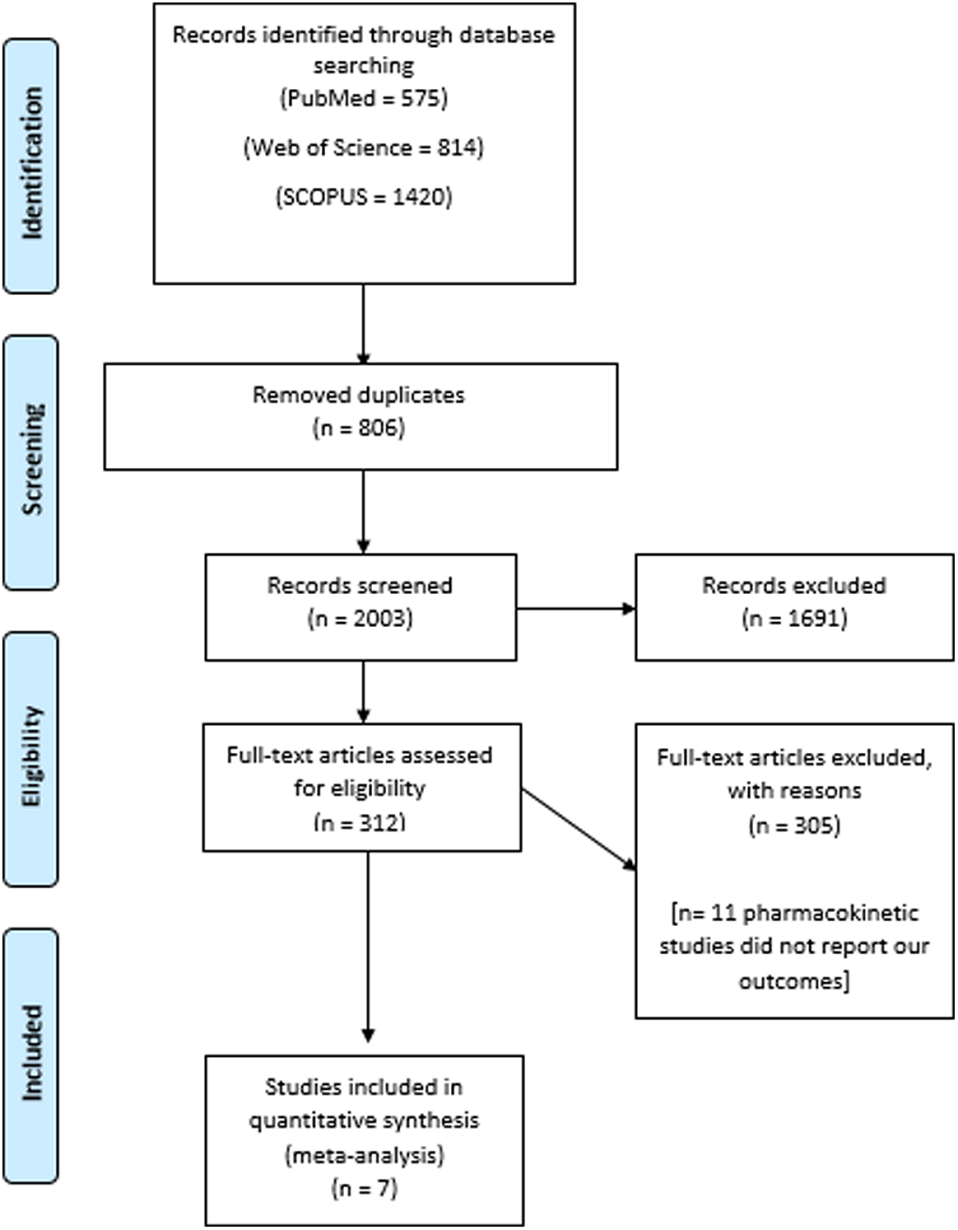
PRISMA checklist.

Figure 1 shows the PRISMA flow diagram of the literature search and included trials. We analyzed data obtained from 999 patients from 7 included trials.^26–32^ Four hundred eighty-nine patients received rifaximin and 510 patients are allocated to the control group. The average age of participants in the treatment group was 53.3 years old, and the mean age of patients in the control group was 52.8 years old; Tables 1 and 2 summarize the data of the included studies and population characteristics.

### Results of Risk of Bias Assessment

**Figure 2.**
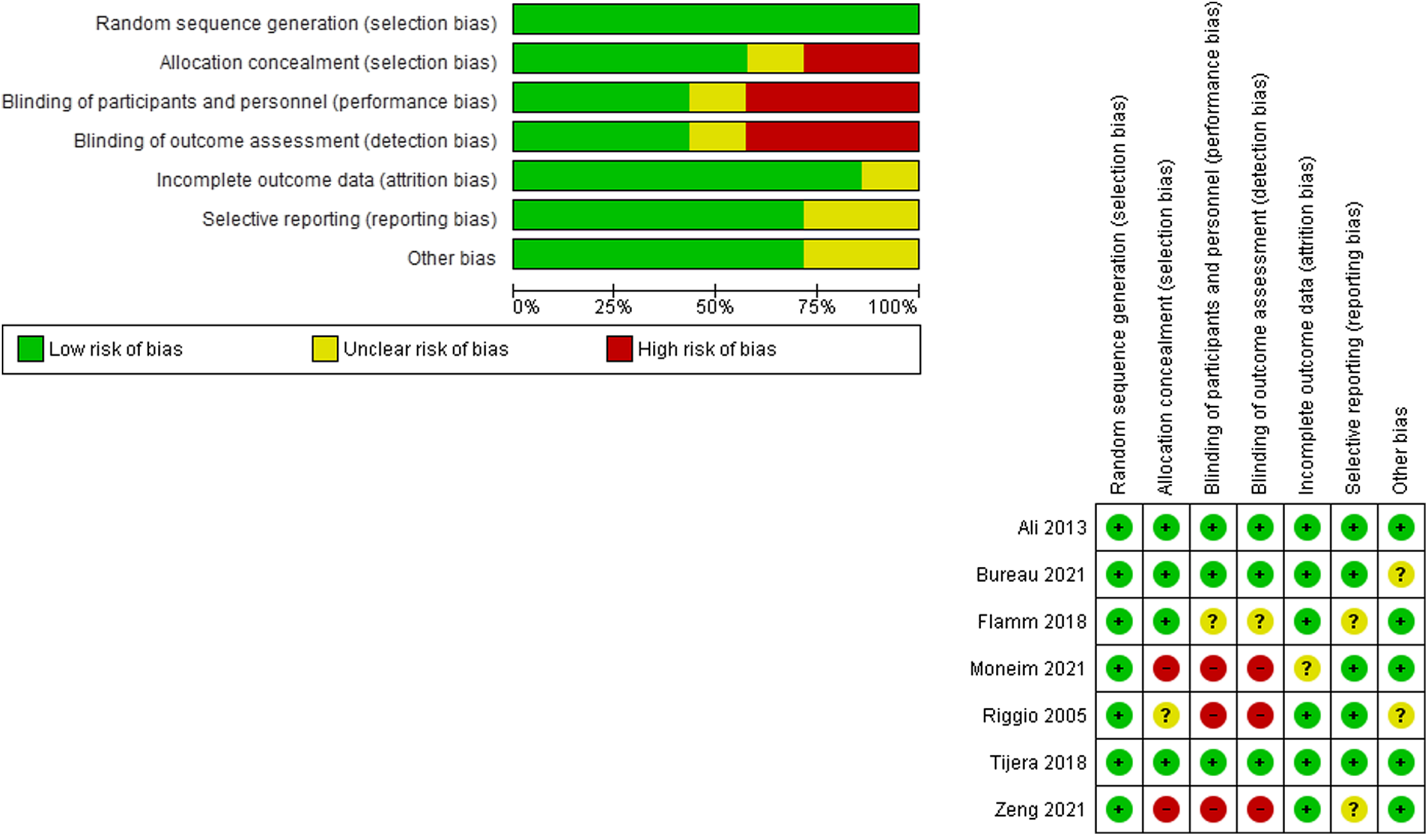
Summary of the quality assessment results.

The combined risk of bias results was low. Figure 2 summarizes the results of the quality assessment of the included studies. Regarding randomization, all studies were at low risk. Regarding allocation concealment, only Riggio et al^27^ did not report enough data and the rest of the trials were at low risk except 2 trials^28,31^ were at high risk. As for blinding of both participants and outcome assessors, 3 studies^27,28,31^ are at high risk of bias and 3 studies are at low risk.^26,30,32^ The attrition bias and selective reporting domains were at low risk of bias in most of the studies. Table 3 shows the summary of the risk of bias results.

### Analysis of Outcomes

**Figure 3.**
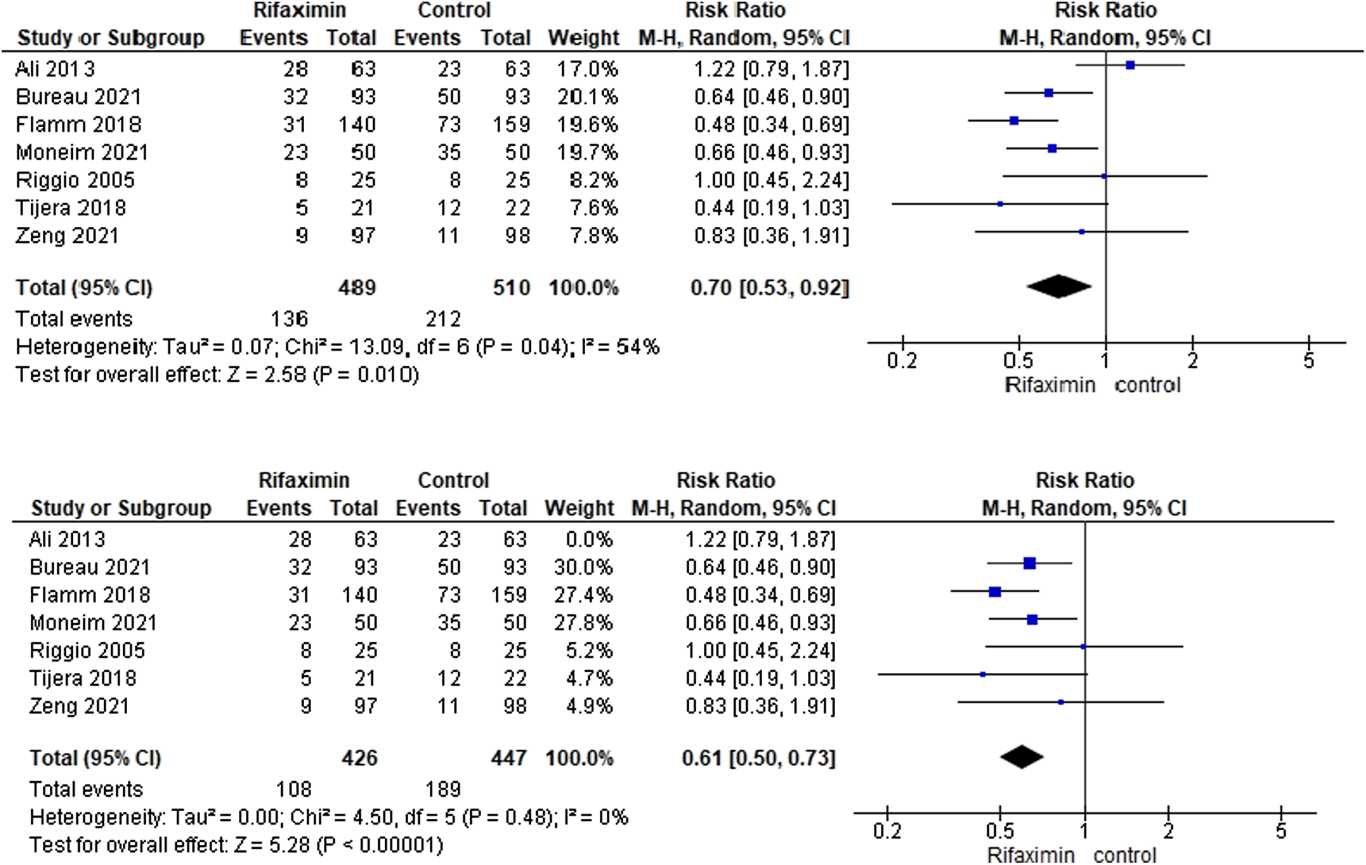
Forest plot of the recurrence of hepatic encephalopathy outcome.

**Figure 4.**
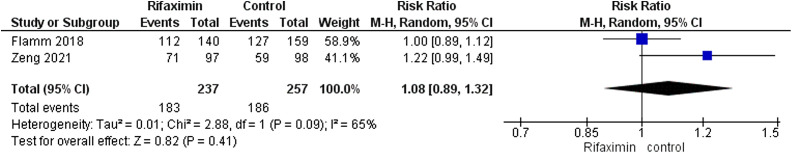
Forest plot of adverse events outcome.

**Figure 5.**
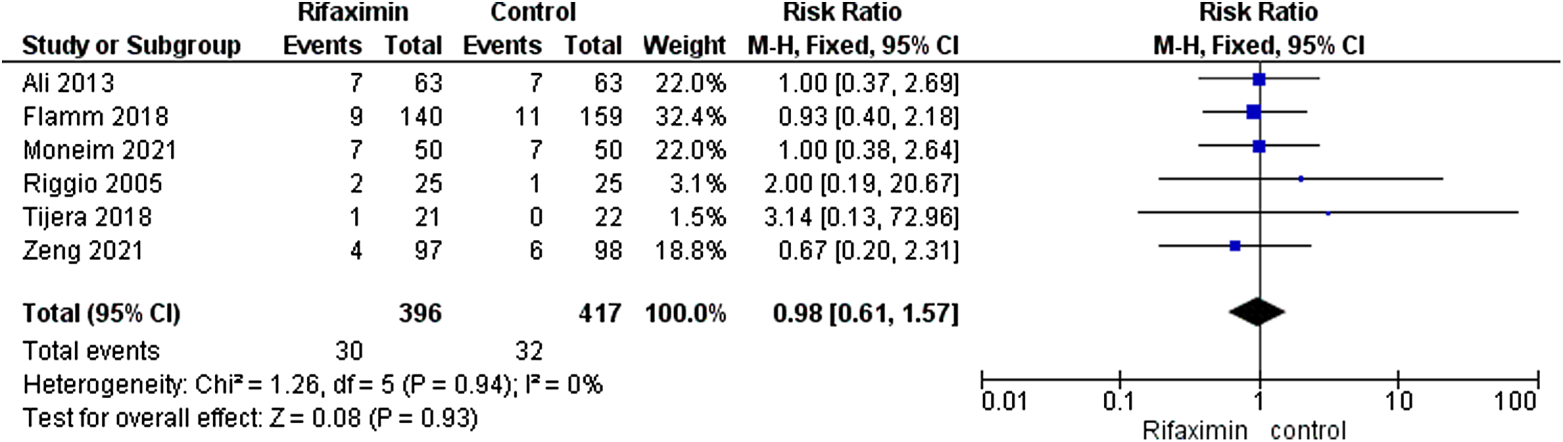
Forest plot of mortality rate outcome.

**Figure 6.**
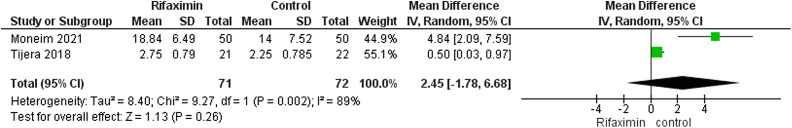
Forest plot of time to the first episode of hepatic encephalopathy from the time of randomization (days) outcome.

1. **Recurrence of HE**
  All the included trials^26–32^ reported this outcome. The pooled analysis showed heterogeneity between the included trials (I^2^ = 54%, *P* =.04, Figure 3A). We solved the heterogeneity by excluding Ali et al.^30^ The overall risk ratio proved that the rifaximin group was associated with a lower recurrence rate than the control group (RR = 0.61[0.50, 0.73], *P* =.001). The pooled analysis becomes homogeneous (I^2^ = 0%, *P* = .48, Figure 3B).
2. **Adverse events**
  This outcome was reported by 2 studies.^29,31^ The overall risk ratio showed a similarity between both groups in the occurrence of adverse events (RR = 1.08 [0.89, 1.32], *P* =.41). The analysis was heterogeneous (I^2^ = 65%, *P* =.09, Figure 4).
3. **Mortality rate**
  A total of 396 patients were analyzed from 6 trials,^27–32^ reporting this outcome. The risk ratio demonstrated that the mortality rate was the same in both groups (RR = 0.98[0.61, 1.57], *P* =.93). The included trials show homogeneity (*I* ^2^ = 0%, *P* =.94, Figure 5).
4. **Time to the first episode of HE from the time of randomization (days)**
  The overall mean difference (MD) proved that the time to the first episode of HE from the time of randomization was not significantly different in both groups (MD = 2.45[−1.78, 6.68], *P* =.26). The analysis was heterogeneous (*I* ^2^ = 89%, *P* =.002, Figure 6).

## DISCUSSION

In this meta-analysis, we estimated the efficacy of rifaximin in preventing the recurrence of HE in patients with hepatic diseases. The meta-analysis showed that the recurrence rate of HE was significantly lower in the rifaximin group than in the control group. However, there was no difference in both groups regarding adverse events, mortality rates, and time to the first episode of HE from the time of randomization.

A randomized trial^31^ of long-term use of low-dose rifaximin can reduce cirrhotic consequences. Cirrhotic complications are the major cause of death in people with end-stage hepatic disease. As a result, avoiding these consequences would considerably enhance the quality of life in cirrhotic patients. The most surprising finding was that rifaximin improved survival in patients with Child-Pugh class C. Although the recommended dose of rifaximin in cirrhosis was 1100-1200 mg/day, this research indicated the ability to employ a low-dose rifaximin maintenance treatment with equal effects.^29,33–36^

Only a few studies reported the effect of rifaximin on reducing the consequences of end-stage hepatic disease and overall survival.^34,36^

The study by Vlachogiannakos et al^34^ involved 23 patients with alcoholic cirrhosis. They reported that 28-day rifaximin with a daily dose of 1200 mg therapy showed improvement in liver hemodynamics, 5-year survival, and reduced vascular complications such as portal hypertension.

Rifaximin is a well-tolerated drug that has a favorable profile. The major reported adverse events were Lyell syndrome and neutropenia which could be resolved completely after symptomatic treatment. In our meta-analysis, the adverse events were similar in both the rifaximin and control groups.

Concerning the prevention of HE in cirrhotic patients with acute variceal bleeding (AVB), Higuera-De-La-Tijera et al^32^ found that anti-ammonium drugs such as rifaximin showed promising safety and efficacy outcomes in terms of primary prevention of HE after variceal bleeding. Although AVB is the second most common cause of HE, there is still no sufficient evidence regarding how to prevent this complication. The trial reported that anti-ammonium drugs decrease the incidence of HE by 25.9% when compared with the placebo. In the same field, Garcia-Tsao and Bosch^37^ studied the effect of lactulose as an anti-ammonium drug on the prevention of HE after AVB. Another risk factor for HE is bacterial infections such as SBP. In the study by Higuera-De-La-Tijera et al^32^ only 3 patients developed SBP, and interestingly, all those patients were not in the rifaximin group. This fact may give attention to performing a well-designed trial to validate the use of rifaximin as prophylactic therapy in SBP.

Flamm et al^29^ investigated the effect of 550 mg BID rifaximin on preventing cirrhosis-related complications based on MELD score, baseline international normalized ratio (INR), and the presence of ascites. They found that rifaximin decreased the risk of first cirrhotic complication compared with placebo. However, the post hoc analysis was still a limitation facing this study. Additionally, data from other studies reported that rifaximin significantly decreased the incidence of acute kidney injury and hepatorenal syndrome compared with no-treatment group.^38^

An open-label prospective study reported that rifaximin had maintained remission from HE in patients with hepatitis C cirrhosis with limited potential to emerge a bacterial resistant through the study period.^28^

Riggio et al^27^ performed the first trial to establish the effect of rifaximin in the prevention of HE after TIPS. The trial was performed in the first month after TIPS which was the period of the highest incidence of HE.^39^ The occurrence of HE was the endpoint of this study.^40^ The following study showed that the use of lactulose (nonabsorbable disaccharide) or rifaximin (nonabsorbable antibiotic) was better than the no-treatment group in decreasing the incidence of HE after TIPS.

This meta-analysis estimated the efficacy of rifaximin in preventing the recurrence of HE in patients with end-stage liver disease. All the included trials were well-designed and the overall risk of bias was low. However, some of these trials were not blinded which was a limitation of this meta-analysis. Schulz et al^41^ examined the 33 meta-analyses including 250 randomized and found that the unblinded trials reported an average odds ratio that was 17% higher than blinded studies. Another limitation was the heterogeneity in some outcomes, we managed to track down the attributing factors and solved the heterogeneity by excluding Ali et al.^30^

## Conclusion

The meta-analysis revealed that the recurrence rate of HE was considerably lower in the rifaximin group than in the control group, with no difference in adverse events or death rates between the 2 groups.

## Figures and Tables

**Table 1.** Summary of the Participants, Their Demographic Data, and MELD Score

Study ID	Sample Size		Age Mean (SD)		MELD Score Mean (SD)		Male (n)%		Female (n)%	
	Rifaximin	Control	Rifaximin	Control	Rifaximin	Control	Rifaximin	Control	Rifaximin	Control
Ali et al^30^	63	63	42.8 (4.54)	40.2 (2.33)	15.45 (3.45)	16.34 (2.87)	31 (49.2%)	29 (46.03%)	32 (50.79%)	34 (53.97%)
Bureau et al^26^	93	93	NR	NR	NR	NR	NR	NR	NR	NR
Flamm et al^29^	140	159	55.5 (9.6)	56.8 (9.2)	13.1 (3.6)	12.7 (3.9)	75 (53.6%)	107 (67.3%)	65 (47.6%)	52 (32.7%)
Abdel Moneim et al^28^	50	50	58.46 ± 7.76	60.50 ± 7.63	NR	NR	30 (60%)	29 (58%)	20(40%)	21(42%)
Riggio et al^27^	25	25	55 (10.8)	54.9 (11.7)	10.7 (6)	8.4 (5.2)	14	21	11	4
Higuera-De-La-Tijera et al^32^	21	22	53.0 (10.9)	49.3 ± 9.5	NR	NR	10 (47.6)	17 (77.3)	11 (52.4)	5 (22.7)
Zeng et al^31^	97	98	56.01 ± 9.34	55.47 ± 9.96	11.30 ± 4.01	11.59 ± 3.58	64 (65.98)	61 (62.24)	33 (34.02)	37 (37.76)

MELD, model for end-stage liver disease.

**Table 2.** Summary of the MELD Score ≤10, MELD Score 11-20, MELD Score 21-25, No. of HE Episodes =2, and No. of HE Episodes >2

Study ID	MELD Score ≤10 (n)		MELD Score 11-20 (n)		MELD Score 21-25 (n)		No. of HE Episodes = 2n (%)		No. of HE Episodes >2n (%)	
	Rifaximin	Control	Rifaximin	Control	Rifaximin	Control	Rifaximin	Control	Rifaximin	Control
Ali et al^30^	2 (3.17%)	5 (7.94%)	34 (53.97%)	35 (55.55%)	27 (42.86%)	23 (36.51%)	30 (47.62%)	25 (39.68%)	33 (52.38%)	38 (60.32%)
Bureau et al^26^	NR	NR	NR	NR	NR	NR	NR	NR	NR	NR
Flamm et al^29^	34 (24.3%)	48 (30.2%)	94 (67.1%)	96 (60.4%)	12 (8.6%)	14 (8.8)	97 (69.3%)	111 (69.8%)	43 (30.7%)	47 (29.6%)
Abdel Moneim et al^28^	7 (14%)	4 (8%)	34 (68%)	33 (66%)	9 (18%)	13 (26%)	NR	NR	NR	NR
Riggio et al^27^	NR	NR	NR	NR	NR	NR	NR	NR	NR	NR
Higuera-De-La-Tijera et al^32^	NR	NR	NR	NR	NR	NR	NR	NR	NR	NR
Zeng et al^31^	NR	NR	NR	NR	NR	NR	NR	NR	NR	NR

HE, hepatic encephalopathy; MELD, model for end-stage liver disease; NR, not reported.

**Table 3.** Summary of Risk of Bias (ROB) of the Included Studies

Study ID	Randomization	Allocation Concealment	Blinding of Personnel and Patients	Blinding of Outcome Assessment	Attrition Bias	Selective Reporting	Other
Ali et al^30^	Low	Low	Low	Low	Low	Low	Low
Bureau et al^26^	Low	Low	Unclear	Unclear	Low	Unclear	Low
Flamm et al^29^	Low	Low	Low	Low	Low	Low	Unclear
Abdel Moneim et al^28^	Low	High	High	High	Unclear	Low	Low
Riggio et al^27^	Low	Unclear	High	High	Low	Low	Unclear
Higuera-De-La-Tijera et al^32^	Low	Low	Low	Low	Low	Low	Low
Zeng et al^31^	Low	High	High	High	Low	Unclear	Low
